# “We're forced to be resilient”: exploration of prospective risk and protective factors of resilience among women athletes

**DOI:** 10.3389/fspor.2026.1718372

**Published:** 2026-03-13

**Authors:** Emily L. Matheson, Kelsey A. Varzeas, Nicole Y. Wesley, Miriam Rowan, Carolyn B. Becker, Kathryn E. Ackerman, Tiffany M. Stewart

**Affiliations:** 1 Behavior Technology Laboratory, Pennington Biomedical Research Center, Baton Rouge, LA, United States; 2 Sports Medicine Division, Boston Children’s Hospital, Boston, MA, United States; 3 Department of Psychology, Trinity University, San Antonio, TX, United States; 4 Sports Medicine, Women’s Health, Sports, and Performance Institute, Boston, MA, United States; 5 Neuroendorine Unit, Massachusetts General Hospital, Boston, MA, United States; 6 Harvard Medical School, Boston, MA, United States

**Keywords:** athletes, early intervention, prevention, qualitative research, resilience, women

## Abstract

**Introduction:**

This qualitative study aims to elucidate findings from a larger research portfolio exploring the prospective risk and protective factors associated with resilience among women athletes.

**Methods:**

Adopting a constructivist approach, semi-structured interviews were conducted with 14 women aged 19–46 years (M = 24 years; SD = 7.64), who represented seven sports and played for an average of 12 years (SD = 5.34). Reflective Thematic Analysis was used to analyze and identify patterns within the data that addressed women's perceptions and experiences of resilience in sport settings, potential barriers and facilitators of resilience, and programmatic preferences for new and/or revised resilience-based trainings.

**Results:**

Three overarching themes and seven subthemes were identified: (1) The system breeds adversity and resilience (sports patriarchy and gender inequality; representation and role models; interpersonal dynamics and environments; resilience has ripple effects); (2) misconceptions and stereotypes of resilience; (3) resilience is a dynamic spectrum of interconnected skills (disconnection to attunement with self and others; avoidance to acceptance of adversity; maladaptive to adaptive coping during adversity).

**Discussion:**

Findings suggest that traditional resilience models do not account for gendered stressors, structural inequalities, and resilience misconceptions embedded within sports cultures. Results support the need for socio-ecologically informed, gender-responsive approaches to research, clinical practice, and athlete support. Future work should integrate quantitative and qualitative methods into a revised resilience theoretical framework, refine resilience measurement, and prioritize adaptive coping strategies within multi-level frameworks to promote sustainable mental health, well-being, and long-term participation in sport.

## Introduction

The extensive mental, physical, and social health benefits associated with sports are well-documented (1–4). However, sports participation and progression also come with inherent risks and challenges, including injury, symptoms of overtraining, complex interpersonal and power dynamics, and potential compromises in other personal and professional domains (5, 6). In addition to these general adversities, women athletes face gender-related systemic biases, inequalities and discrimination that create additional burdens and put them at greater risk of poor health outcomes, including anxiety, depression, relative energy deficiency in sport (REDs), eating disorders, and suicidal ideation relative to men (7–11). Common gender biases, inequalities and discrimination faced by women in sport include poorer-quality apparel, equipment, and infrastructure (12), lower financial investment and remuneration (13), limited and objectifying media coverage (14), overevaluation of appearance and body shaming (15), limited playing, occupational, and leadership opportunities (12), and increased harassment and abuse (16). In light of these additional burdens and stressors, a small but emerging body of work has explored resilience exclusively among women in sport (17).

The term *resilience* has been widely researched among different populations, including cancer survivors, persons with chronic health and disabilities, healthcare providers, military personnel and veterans, and trauma survivors (18–22). Resilience research among athletes emerged in the early 2000s, with mixed-methods research leading to several theoretical frameworks that aim to explain how athletes come to develop and maintain resilience, including the Conceptual Model of Sport Resilience (23), the Grounded Theory of Psychological Resilience and Optimal Sport Performance (24), and the Stress-Resilience-Performance Model (25). Much of the research underpinning these models, and the studies that ensued, has been conducted among mix-gender samples, and did not consider the unique gender-related variables associated with, and impacting, women's resilience in sport.

In a recent review, O'Brien and colleagues (17) defined and described resilience as it pertains to girls and women in sport and provided points for consideration and suggestions for bolstering resilience among these populations. A key suggestion was the use of existing mental skills training and psychological interventions. Although these approaches show some promise at improving variables associated with resilience (e.g., problem solving and mindfulness), there is little support for their efficacy on validated resilience measures. Further, the proposed approaches by O'Brien and colleagues (17) were tested among mixed-gendered athlete samples, and do not account for gender-related variables and stressors. Only two studies have evaluated resilience-based interventions exclusively among women athletes (HeartMath®) (26); (Pressure Training Intervention) (27), with only one of the studies evaluating changes in resilience (26). Intervention efficacy was not established by Miller and France (26); however, study limitations, including a small sample size (i.e., *N* = 7), limit the robustness and validity of their conclusions. Given these research patterns and limitations, there is cause to revisit the empirical data that underpin resilience theories pertaining to women athletes and the subsequent interventions selected for and applied to this population.

This qualitative study is part of a broader research portfolio exploring prospective risk and protective factors for resilience among women athletes, with the goal of informing resilience-based training interventions. The portfolio employs a mixed-methods approach, including two quantitative studies (28, 29). The first quantitative study established baseline correlations between resilience and potential risk and protective factors. These findings informed the second quantitative study, which examined longitudinal relationships between upstream and downstream variables and resilience. For example baseline emotion regulation difficulties, experiential avoidance, intolerance of uncertainty, social support, and sleep difficulties, all significantly predict decrements in one or both measures of resilience longitudinally, thus confirming that these constructs may serve as prospective risk factors for decreased resilience in female athletes (29). The second study also reinforced that higher resilience significantly prospectively predicted improved mental health outcomes among female athletes (29).

This qualitative study aims to bridge the gap between numbers and narratives by exploring women athletes' understanding and experience of resilience in sport settings. The addition of qualitative research provides a more comprehensive and nuanced conceptualization of resilience and allows for the exploration of emerging themes and variables not necessarily captured by quantitative methods alone. As this research uses a qualitative approach, hypotheses were not generated *a priori*. Instead, the study was guided by three overarching aims that were guided by a constructivist and socioecological understanding of resilience as a dynamic, contextually embedded process shaped by interactions between individuals and their environments (30):

1. Explore women athletes' perceptions and experiences of resilience and compare these between those who self-report as low and high on resilience measures
2. Identify barriers and facilitators to developing and maintaining resilience in women's sport; and
3. Determine athlete's programmatic preferences for a resilience-based intervention.

## Materials and methods

### Participants and procedure

Following Institution Review Board (IRB) ethics approval at Pennington Biomedical Research Center, athletes were recruited via social media posts and snowball sampling using the research team's professional network (e.g., sports teams at universities with which researchers have connections). Recruitment materials explicitly emphasized the aim to represent diverse voices and experiences among women athletes. Participants from underrepresented groups, including athletes of color and those identifying as LGBTQIA+, were encouraged to participate. Outreach communications also highlighted the importance of recruiting athletes across varied ages, racial and ethnic identities, sexual orientations, and sport types, given the intersecting factors that shape resilience experiences.

All recruitment correspondence and materials contained a survey link for participant information, consent, and screening. Initially, eligible athletes were women aged 18 years and over, who scored in the lower (0–29) or upper quartiles (37–40) of the Connor-Davidson Resilience Scale (CD-RISC) and participated in a sport for a minimum of 10 h per week. During the recruitment phase, the majority of the initial interested participants fell into the low-resilience category causing a skewed sample of participants from the lower quartile. To ensure perspectives of both high and low resilience athletes were present, the research team expanded eligibility of the upper quartile from 37 to 40 to 35–40 with all other eligibility criteria remaining consistent. If eligible and consenting, participants provided their email address for follow-up communication with the research team. Participants were contacted within 24 h of completing the survey.

A semi-structured interview guide was developed to broadly discuss athlete's understanding of resilience in women's sports. The development of the guide was an iterative process, beginning with a template designed by the research team involved in the parent quantitative study. The first and second authors made extensive revisions, including refining questions to ensure sensitivity to potentially vulnerable topics (e.g., word usage, cultural considerations, and technical language) and to avoid leading language that might influence participants' responses. For example, the original guide included terms such as “adversity” and “bouncing back,” both of which may carry cultural assumptions about what constitutes resilience. To create a softer, more conversational guide that was open to a variety of perspectives, these terms were revised to “challenges” and “resilience,” thereby reducing potential assumptions and leading phrasing. The questions were designed to allow athletes to reflect on their perceptions of and experiences with resilience, as well as biopsychosocial factors influencing women's resilience in sport. Participants were encouraged to reflect on past athletic experiences and difficult situations, allowing for retrospective insight into how resilience was understood and enacted across different points in their athletic careers. No questions focused on resilience outside of athletic environments.

With continued refinement, the final interview guide included four main sections: (1) Welcome; (2) Athletes' Understanding of and Experience with Resilience; (3) Barriers and Facilitators of Resilience; and (4) Potential Programmatic Features. Sections 2–4 consisted of open-ended questions structured around one overarching question supplemented by two to four follow-up prompts. In total, there were 20 questions across these sections. To assess clarity and test procedures, the interview guide was piloted with two athletes (31). Two participants were deemed appropriate for piloting, as the aim was refinement rather than data saturation (31). These pilots were not included in the final sample due to modifications to question wording and interview structure. Following completion of the pilot interviews, the research team determined that the guide adequately addressed the research aims. Sample questions from the final guide include: *How would you describe the word resilience to someone who hasn't heard this word before? What happens when an athlete isn't resilient in sport? Who or what makes it easier for women athletes to be resilient in sport? Would you consider yourself a resilient person, and can you explain why or why not?.*

Following consent and screening, participants were randomized to speak with one of two interviewers (first and second authors) for approximately 60 min on Zoom. The first author identifies as a White, Australian woman in her early thirties and is a former athlete with 10 years of research experience. The second author identifies as a White, American woman in her early thirties and is a former athlete with 6 years of research experience.

### Data analysis

The analyses were conducted by the first and second authors, who regularly consulted with the authoring team. The dataset was split for analysis between the two authors for efficiency (see Table 1 for assignment). Each interview was transcribed verbatim using Microsoft SharePoint software, and the first and second authors reviewed each transcript for accuracy. Following the transcription process, Reflective Thematic Analysis was manually conducted (32–34).

**Table 1:** Participant pseudonyms and demographics.

Participant Number & Pseudonym	Interviewer & Analysist	Age	Ethnicity	Sexual Orientation	Current Occupation	Resilience Score & Quartile	Primary Sport	Years in Primary Sport	Current Competition Level	Highest Competition Level
P1—Harper	A2	22	White	Heterosexual	College Senior	27—L	Rowing	3	Collegiate	Collegiate
P2—Alice	A1	21	White	Heterosexual	College Senior	40—U	Soccer	15	Collegiate	Collegiate
P3—Carly	A1	29	White	Lesbian/Bisexual	Professional soccer player	29—L	Soccer	18	Professional	Professional
P4—Olivia	A1	30	White	Heterosexual	Psychologist	24—L	Boxing	10	Recreational	Collegiate
P5—Maria	A2	46	White	Heterosexual	Athletic Coach	29—L	Triathlon	20	Recreational	Professional
P6—Casey	A2	22	White	Lesbian/Gay	Graduate Student	37—U	Ice Hockey	18	Collegiate	Collegiate
P7—Eloise	A1	19	White	Heterosexual	College Junior	28—L	Ice Hockey	14	Collegiate	Collegiate
P8—Sam	A2	32	White	Heterosexual	Nurse Anesthetist	29—L	Triathlon	1.5	Recreational	Collegiate
P9—Eliza	A2	21	White	Heterosexual	College Junior	25—L	Ice Hockey	15	Collegiate	Collegiate
P10—Courtney	A1	20	Native Hawaiian or Other Pacific Islander	Heterosexual	College Sophomore	37—U	Ice Hockey	14	Collegiate	Collegiate
P11—Ava	A2	24	White	Heterosexual	Graduate Student	24—L	Volleyball	15	Collegiate	Collegiate
P12—Hannah	A1	19	Asian	Heterosexual	College Sophomore	28—L	Ice Hockey	14	Collegiate	Collegiate
P13—Meredith	A2	19	Asian	Heterosexual	College Sophomore	40—U	Lacrosse	9	Collegiate	Collegiate
P14—Gabby	A1	18	Mixed Race	Heterosexual	College Freshman	27—L	Ice Hockey	12	Collegiate	Collegiate

Initially, the authors read and re-read their assigned interviews and took notes of preliminary observations and ideas. An initial coding framework was developed based on two interviews; an athlete with a lower quartile score (**Eloise**) on the CD-RISC, and the other with an upper quartile score (**Casey**). To ensure inter-rater reliability during split-coding and analyses, both authors analyzed the same first two interviews. Separately, the authors generated and defined codes and extracted corresponding quotes; the authors then reconvened to align on discrepancies and finalize the framework, which would then be applied to the remaining 12 interviews.

Following Eloise's and Casey's interviews, the first and second authors conducted three rounds of coding, whereby they coded two interviews each, totaling four interviews per round. After each round, the authors discussed each interview, reviewed newly generated or redundant codes, and finalized the framework ahead of the next round. After the fourth round, codes were finalized, and the authors identified similarities and discrepancies to generate and define unifying themes and subthemes and identified exemplary quotes. Finally, the first and second authors wrote the results section, whereby they further refined the findings, and considered new ideas that emerged during the writing process.

The current project's research paradigm was guided by a social constructivism approach, where realities are constructed through personal experiences and knowledge is created through interactions with others and with the environments the participants live in (35, 36). Important to this research paradigm is the ongoing construction of participant reality with others and within the broader social, cultural, and historical contexts in which they reside. With regards to the current study, participants' perspectives on resilience as a woman athlete were not created in isolation, but rather, formed by shared experiences and cultural contexts. As such, the first and second authors took an interpretivism approach to this work honoring the participants' knowledge as subjective, socially constructed, and created through understanding the meanings and experiences of individuals (37).

## Results

As with all qualitative research, the retelling of stories involves a questioning voice, clarity, and interpretation of narrative experiences causing meaning-making to be a vital interplay between the participant and the researcher (38). In the process of telling one's story, participants are the first interpreters of their experiences and constructors of their realities with the role of the researcher as a supporter of the meaning-making process (38). Therefore, in sharing the experiences of these participants, the researchers have committed to keeping perspectives as intact as possible, acknowledging that re-assembling in the analysis process may require re/presenting events with modifications.

Due to time and length of participant interviews, perspectives shared could not be kept entirely in the participants' own words. Direct speech in the form of quotes is presented using italics rather than quotation marks, as the participants have not been quoted exactly. Extraneous words (e.g., “um” or “like”) and repetition have been deleted, and certain words have been omitted to ensure confidentiality. Importantly, there is no absolute truth about how something is interpreted (39). Qualitative methodology uses context, individual experience, and subjective interpretation, by which generalizability is not possible and not the goal (40). As researchers, we respect that other investigators would have made different re/presentations based upon their own worldviews. Importantly, these stories are not character studies of participants or classifications of high and low resilience but glimpses into circumstances in certain contexts.

Pseudonyms and demographics for the fourteen athletes are reported in Table 1. Participants had a mean age of 24 years (*SD* = 7.64; range = 19–46 years), with a majority identifying as White (71.5%) and heterosexual (85.7%). Of the remaining participants, 14.3% identified as Asian, 7% as Mixed Race, and 7% as Islander with 14.3% identifying as non-heterosexual. On average, participants played sport for 12 years (*SD* = 5.34; Min = 1.5—Max = 20), and represented seven different sports including boxing, ice hockey, lacrosse, triathlon, rowing, soccer, and volleyball. Of the 14 participants, 10 scored in the lower quartile on the CD-RISC (0–29) and four scored in the upper quartiles (35–40). The participants' pseudonyms, resilience quartile (LQ = lower quartile; UQ = upper quartile), age, and sport are provided in full upon their first citation, with abbreviations used in subsequent quotes, thus further contextualizing the data within the respective themes [e.g., Eloise (LQ; 19; Ice Hockey)]. Participants represented a range of athletic contexts, including current collegiate athletes, retired collegiate athletes, recreational competitors, and professional athletes. While the majority competed in team-based sports (e.g., ice hockey, soccer, volleyball), several participants represented individual sports (e.g., triathlon, boxing, rowing). These contextual differences shaped how adversity was experienced and navigated and are noted throughout the themes where relevant.

The interviews took an average of 50 min (*Min* = 33; *Max* = 64), and following Reflective Thematic Analysis, four themes and seven subthemes were identified. The first theme, *The System Breeds Adversity and Resilience*, encompassed four related dimensions: sports patriarchy and gender inequality; representation and role models; interpersonal dynamics and environments; resilience has ripple effects. The second theme, *Misconceptions and Stereotypes of Resilience*, stood alone without further subdivisions. The third theme, *Resilience is a Dynamic Spectrum of Interconnected Skills*, comprised three continuums: disconnection to attunement with self and others; avoidance to acceptance of adversity; and maladaptive to adaptive coping during adversity. The final theme, *Programmatic Preferences*, centered on programmatic features and considerations for implementation.

Although themes were generated inductively, findings reflect influences operating across multiple levels of the sporting ecosystem (41, 42). Specifically, participants described societal and cultural forces (e.g., gender inequities), organizational structures (e.g., institutional hierarchies and resource allocation), interpersonal dynamics (e.g., coach-athlete relationships), and individual-level processes (e.g., coping and self-regulation) as shaping resilience. Themes are presented as identified in analysis, with this multi-level structure used to orient interpretation, and Table 2 provides a concise summary of perceived risk and protective factors across these levels.

**Table 2:** Perceived protective and risk factors influencing women athletes' resilience across the socio-ecological model (41, 42).

Socio-Ecological Level	Primary Theme and Subtheme Alignment	Protective Influences	Risk & Undermining Influences
Societal & Cultural	**T1:** The System Breeds Adversity and Resilience ◦ **ST1:** Sports patriarchy and gender inequality; ◦ **ST2:** Representation and role models **T2:** Misconceptions and Stereotypes of Resilience	Growing media visibility Recognition of inequities Equity advocacy Access to women's sport stories Social media exposure Reframing resilience Self-compassion Patience; growth-oriented mindset	Gender inequity Patriarchal sport norms Unequal pay Resource disparities Scheduling inequities Historical underrepresentation Limited role model visibility “Bounce back” narrative Resilience equated with suffering Shame; quitting framed as weakness
Organizational & Institutional	**T1:** The System Breeds Adversity and Resilience ◦ **ST1:** Sports patriarchy and gender inequality ◦ **ST3:** Interpersonal dynamics and environments	Structured, integrated support systems Institutional psychological safety Equitable staffing Mental health resources Athletic trainers	Unequal funding Limited staff Rigid hierarchies Performance-driven climates Limited mental health access
Interpersonal	**T1:** The System Breeds Adversity and Resilience ◦ **ST3:** Interpersonal dynamics and environments ◦ **ST3:** Resilience has ripple effects	Emotionally intelligent and self-aware coaches Positive peer modelling Parental encouragement Open dialogue Collective motivation Shared accountability	Dismissive coaching Favoritism Mental health stigma Harmful team culture Normalization of suffering
Individual	**T3:** Resilience is a Dynamic Spectrum of Interconnected Skills ◦ **ST1:** Disconnection to attunement with self and others ◦ **ST2:** Avoidance to acceptance of adversity ◦ **ST3:** Maladaptive to adaptive coping during adversity	Self-awareness Emotional regulation Internal locus of control Relational connection Injury acknowledgment Feedback openness Mindfulness Adaptive reframing and self-talk Problem solving Help-seeking	Emotional suppression Social withdrawal Self-criticism Comparison making Catastrophizing Injury denial Resistance to feedback Overtraining Premature return

T, theme; ST, sub-theme.

### The system breeds adversity and resilience

Each athlete identified and described how sport systems, whether organizational (e.g., an athletic department), institutional (e.g., National Collegiate Athletic Association), or societal (e.g., representation of women in sports media), inherently create challenges and obstacles for women, requiring them to develop and maintain resilience.

#### Sports patriarchy and gender inequality

Most participants acknowledged that sports systems were designed for and favored men, and that harmful gender stereotypes and biases are deeply engrained and normalized in sport culture. **Carly** (LQ), a 29-year-old professional soccer player with 24 years of playing experience, explained:

*We're forced to be resilient, because throughout our entire lives we're constantly compared to men and deemed inferior. They're seen as worth more than us, funnier than us, smarter than us, and more athletic than us…They also have so many different avenues to succeed. You [women] must be that much more of the.0001% to be able to make it, or you need to be willing to make a lot of sacrifices.* Similar sentiments were expressed across competition levels, including by collegiate athletes competing within NCAA structures, suggesting that gendered inequities were perceived as systemic rather than competition-level specific. There was also a consensus among participants that women are expected to perform and succeed within their sport, with significantly fewer resources at their disposals. For example, a common observation was discrepancies in resource investment and allocation between women's and men's teams. For example, **Eliza** (LQ), a 21-year-old collegiate ice-hockey player reported:

It's hard to see guys getting more stuff or being treated better for no real reason. Even little things like clothes and free stuff—they get a huge pile, and we don't. Their games are at 7:00PM when everyone can come, while ours are at 2:00PM in the middle of the school day. They have better photographers, videographers, and announcers. It's frustrating because we do the same work they do, yet they get more. It makes you want to stop trying because why put in the same effort when they're treated so much better?

Throughout the interviews, participants acknowledged the subpar sport conditions for girls and women, and how they were required to make sacrifices to not only succeed but to simply participate. Participants mentioned a lack of playing and professional opportunities, unequal pay, using personal finances to cover equipment and apparel, and relocating away from support networks to pursue opportunities. These inequalities force women to work exponentially harder than their male counterparts, making success an exception rather than the norm.

#### Representation and role models

Participants emphasized that representation was not simply about visibility, but about meaningful access to women athletes' stories and lived experiences. Learning how other women navigated adversity was described as imperative to building and sustaining resilience. Historically, women's sport has been underrepresented in mainstream media, limiting opportunities for identification and vicarious modelling. However, several participants acknowledged that coverage has been “*increasing*” and “*improving*”, and that social media has become a gateway for connecting with past and present athletes who were previously less visible. As explained by **Harper** (LQ), a 21-year-old collegiate rower:

When you're little, your parents have to jump through seven hurdles for you to watch the game—that's what it was like for us to watch the WNSL for a long time. Social media made it so easy to find support, particularly Instagram and YouTube. I would watch YouTube videos that were published in 2007 and rewatch those. That was huge.

Numerous athletes discussed more proximal environments, emphasizing how direct contact with positive role models not only humanized common adversities, such as injuries, difficult team cultures, and gender inequality, but also provided opportunities for advice and modelling for how to work through these inevitable challenges. **Carly (LQ; 29; Soccer)** recalled that when she was 14 years old, Abby Wombach visited her soccer team while recovering from a severe broken leg, which prevented Wombach from playing in the 2008 Beijing Olympics. Carly acknowledged that while it was amazing to meet her “*idol”*, it was more impactful “*to continue to watch her play, set records and score goals after she came back from the break—I thought, anything's possible.”*

Throughout the interviews, participants expressed a strong desire to learn about other women's sports stories, recognizing that this visibility broadened their understanding of what is possible, alleviated their concerns about the unknown, and boosted their confidence in navigating adversities. Further, some participants emphasized that representation does not only involve celebrating women's athletic successes but also sharing the full spectrum of their sport experiences. By doing so, it shines light on possible social injustices and inequalities, fosters relatability and connection among players, and inspires and empowers athletes to reach new heights.

#### Interpersonal dynamics and environments

When asked about what factors make it easier or harder for athletes to be resilient, most athletes were quick to identify and discuss the role of coaches. Coaches were perceived as either a great resource or hindrance to an athlete's overall health, wellbeing, and performance, with athletes typically recalling more negative coaching experiences than positive. Athletes described coaches who were “*unapproachable*”, “*non-communicative*”, “*performance-driven*” and engaging in “*favoritism*” and how these approaches were antithetical to building resilient athletes and team cultures. Notably, athletes in team-based collegiate environments described more formalized hierarchies and daily exposure to coaching staff, whereas those in individual or recreational sports described less structured oversight; however, across contexts, coaches were perceived as powerful facilitators or barriers to resilience.

As explained by **Olivia** (LQ), a 30-year-old former collegiate field hockey player and now boxer: *I see it often with unhelpful coaches who push you to do more and give more, even when you're not feeling your best. This can lead to serious, even career-ending injuries. They also tend to dismiss mental health, either by discouraging you from seeing a therapist or penalizing you if you do—like benching you or not taking you seriously anymore.*

On the other hand, when reporting on the type of relationships that bolstered athletes' resilience, a broader support network was described. As **Harper (LQ; 21; Rower)** noted: *At an institution, having athletic trainers who give you the space to acknowledge your pain and seek help is invaluable. Equally important are mental, physical, and nutritional support systems, along with parents, friends, and coaches.*

In addition to identifying *who* made up these positive support networks, participants described *how* individuals within these networks enhanced their resilience. Common traits of these individuals included: acknowledgment that athletes are multifaceted, valuing of vulnerability, encouragement of open and constructive dialogue, and a commitment to creating a culture of mutual respect, safety, understanding, and growth. This was reflected in 22-year-old **Casey's** (UQ) account of a three-month recovery from a collegiate ice hockey injury:

I'm thinking a lot about the sport environment; the hierarchy of coaches and captains…If I had been in a more toxic sporting environment, I wouldn't have healed the way I did. My coaches [and teammates] were incredibly supportive throughout the process, and I felt comfortable bringing my negative feelings to my athletic trainer and psychiatrist, even at my worst.

Meanwhile, **Maria** (LQ) a 46-year-old triathlete, spoke to the role of parents in supporting athlete's resilience, recounting how her father's parenting style had a long-lasting impact on her approach to adversity, both in sport and life more broadly:

I was raised in an environment that valued pausing, being mindful, and appreciating the moment, while also prioritizing movement and mental growth. My dad always said, “It’s not about the fall, but how you get up afterward,” and he truly lived by that. He emphasized learning to “fall upward,” and that process, whatever it looks like, will make you a better person. That mindset was instilled in me so early that, even if I lost sight of it at times, it has always remained a constant in my life.

Overall, participants described interpersonal dynamics as powerful influences on resilience, with coaches occupying a particularly central and often ambivalent role. While coaches were frequently identified as significant barriers to resilience, they were also positioned as primary sources of guidance and support within constrained sport systems. Participants recognized a range of positive influences, including athletic trainers, teammates, and family members; however, these resources were not consistently available. Gender disparities in women's sport, such as limited investment in staffing and infrastructure and the need to relocate to pursue opportunities, often restricted access to broader and stable support networks. Consequently, coaches frequently became the primary, and at times sole, source of interpersonal influence. This concentration of responsibility both heightened the impact of coaching behaviors on resilience and limited athletes' exposure to diverse perspectives, shaping resilience development in ways that were deeply embedded within the structure of the sport environment.

#### Resilience has a ripple effect

Participants perceived resilience as a critical component of thriving sport teams, and considered resilient players and staff invaluable assets, as their attitudes and behaviors “*filter through*” individuals, teams, and environments. As described by **Courtney** (UQ), a 20-year-old collegiate ice-hockey player: *Even if it's one person that brings resilience to a team, if they are bringing that power and mentality, it can be a game changer*.

When recalling characteristics of resilient people within their immediate environments, participants described these individuals as “*positive*”, “*persevering*”, “*inspiring”* and “*motivating*”. **Hannah** (LQ), a 19-year-old collegiate ice-hockey player, elaborated on how these characteristics bettered her and her teammates: *Being around resilient people helps my resilience because it just makes me want to be better for them. It makes me want to show up and be like, “Hey, I need to be the best—not just for myself, but so other people on the team can thrive and get what they want out of it as well.” Because if one person goes down, the rest of us will follow.*

Taken together across these subthemes, participants described sport systems as simultaneously generating adversity and shaping the conditions under which resilience develops. Historically male-dominated structures were perceived to normalize inequities and impose additional burdens on women athletes. At the same time, increased representation and access to role models were viewed as critical in expanding what felt possible and providing tangible examples of navigating adversity. Supportive and psychologically safe environments, particularly those fostered by coaches, staff, teammates, and family members, were identified as central to resilience development. Finally, resilience was understood as relational and contagious; witnessing others persist through challenges reinforced collective confidence and contributed to cultures of perseverance and growth.

### Misconceptions and stereotypes of resilience

Some participants acknowledged that resilience has become a “*buzzword*” and that it was common practice for the term “*resilience*” to be featured in team norms and rules; however, they noted discrepancies in people's understanding and promotion of this concept. On several occasions, participants cautioned that some teammates, staff, and environments promoted inaccurate, oversimplified, and harmful definitions of resilience, which negatively affected their self-beliefs and perceived ability to cope. For example, **Maria** (LQ; 46; Triathlon) noted an expectation that athletes should inherently be resilient but that there was a lack of support for developing the necessary skills associated with resilience.

Sometimes there's shame associated with not being resilient, as if we’re simply expected to be resilient all the time. *There is a narrative that you just bounce back, but there's not a lot of focus on the “how” and the “why”, and the support needed to be resilient.*

In some instances, participants conceptualized resilience as the number of adversities they had endured rather than the skills developed to navigate these challenges or the outcomes achieved. For example, when explaining why she perceived herself as resilient, **Carly** (LQ; 29; Soccer) recalled:

*I'm definitely a resilient person. I've overcome three knee surgeries that kept me out for years—surgeries that were considered career-ending and probably should have been, but I pushed through. I've dealt with medical issues, seizures, concussions, depression, and mental health challenges—just about anything you can think of. At any given point in my life, I've been facing and overcoming something. So yes, I'd say I've endured a lot to get to where I am.* Whereas **Alice** (UQ), a 22-year-old collegiate soccer player, explained how her understanding of resilience has evolved from a stereotypical and potentially harmful definition to a more holistic and adaptive stance:

The stereotypical definition of resilience is the idea of plowing through anything that comes your way. Four years ago, I might have agreed with that. But now, I see resilience differently—it's about staying the course and recognizing that forward progress doesn't always have to be fast. Slow progress is still progress. Overcoming challenges, which others might call resilience, isn't an overnight process. Resilience also means having patience and practicing self-compassion along the way.

A key difference between these two conceptualizations of resilience lies in the emotive and ruminative nature of Carly's description compared to Alice's pragmatic, future-oriented and skills-focused perspective. Carly who was competing at the professional level at the time of interview, centered her athletic identity around prolonged exposure to elite-level physical adversity, offering an extensive account of hardships including career-threatening injuries. In contrast, Alice, alongside other younger collegiate athletes, framed resilience as an evolving journey that, emphasized psychological and behavioral processes for navigating life effectively, rather than as a reflection of accumulated suffering or severe adversity alone.

Another common finding among participants was the association between quitting and a lack of resilience. Across numerous interviews “*quitting*,” “*giving up*,” and “*walking away*” were used synonymously and often described as characteristics of low resilience. For example, **Carly** (LQ; 29; Soccer) reflected on the challenges she had faced in her career, stating: *If I didn't have resilience, maybe I would have been like, “Ah, you know what? I quit*” *or* “*This just isn't for me”. It's not what I want. I'm going to do something else that will satisfy me in other ways.*

While perseverance was often seen as a key aspect of resilience, some participants noted that not all athletes had equal access to the resources and support needed to sustain it and continue in sport. They highlighted how systemic barriers, particularly for girls and women, hindered long-term participation. Many athletes described a lack of support structures that led to early disengagement from sports. As **Courtney** (UQ; 20; Ice Hockey) noted:

I think it's junior year of high school; there's a statistic that shows that most girls quit their sport by junior year. In high school, I see a big issue with coaches not providing their players with the right resources, support, or what they need to be successful and grow to play at the next level.

Participants challenged common assumptions about resilience, particularly the expectation that athletes are inherently “*supposed to be*” resilient. Many emphasized the belief that overcoming hardship is often seen as essential to building resilience, reinforcing the idea that struggle is a necessary part of personal growth. However, this perspective can overlook the complexity of individual experiences, as disengaging from challenges is frequently perceived as failure or a lack of resilience rather than a strategic or self-preserving choice. These findings highlight the need to clarify resilience in sports, recognizing that stepping away from adversity can sometimes be a deliberate and necessary decision rather than a sign of weakness.

### Resilience is a dynamic spectrum of interconnected traits and skills

When reflecting on the individual traits, characteristics and skills associated with resilience, participants' descriptions suggested that resilience is not a fixed quality but rather a dynamic, context-responsive process shaped by a spectrum of interconnected traits, and skills that interplay and collectively support or hinder an athlete's ability to adapt, recover and grow through adversity over time.

#### Disconnection to attunement with self and others

Numerous participants spoke to the spectrum of disconnection and attunement with their bodies, emotions, and support systems. Participants made a direct link between disconnection and a difficulty to respond effectively to stress and/or adversity, while attunement was linked to a deeper understanding of themselves, allowing for healthier decision making and stronger relationships. Disconnection manifested as ignoring or not registering bodily cues, emotion dysregulation, and unhelpful thinking patterns (i.e., all or nothing thinking, catastrophizing, comparison making and fortune telling), which distorted reality, particularly with regards to athletic performance and perceived social evaluations of others, and reinforced negative emotions. As explained by **Eloise** (LQ; 19; Ice Hockey):

I'd say comparison was a big challenge for me [in Freshman year]. When I got into a negative headspace—thinking, 'She's better at that than me'—it was hard to move past. I'd hyper-focus on that one thing and forget about all the other strengths in my game that had helped me get to where I was. I also struggled with staying quiet and isolating myself when things got tough. If I was upset about my performance, I'd withdraw instead of leaning on the people around me. I’d forget to enjoy the game because I was too focused on not performing well, which left me feeling angry and frustrated. Instead of thinking, “Forget about it, it was one shift—flip it, start fresh,” I found it hard to reset, trust in my abilities, and stay in the moment.

Alternatively, some participants described a deeper connection with their own thoughts, feelings, and behaviors, as well as with the people around them. This attunement was expressed through introspective reflection, internal locus of control and a recognition of shared struggles and experiences among athletes. Participants described an iterative process of balancing effort with recovery. This typically involved checking in with themselves, identifying “*controllable”* vs. “*uncontrollable”* factors and deciding on a course of action that is in the best interest of not only them but their teammates, coaches, friends and family. **Maria** (LQ; 46; Triathlon) recalled what introspection looked and sounded like for her:

I think I am pretty good at being resilient. Obviously, it is a learned skill, but I’ve gotten better at asking myself: “Why are you thinking that?”, “Why are you saying that?” or “Why are you doing this?” Then I say to myself, “Hold on”. Thinking like this isn't productive. What do I need? What can I do to neutralize it a little bit and then keep going?

Similarly, while sharing an injury experience, **Olivia** (LQ; 30; Boxer) identified a shift in her thinking and behavior, from an initial place of dwelling and helplessness toward a recognition that she had ownership over her response and could influence the outcome:

I went through all the emotions about my injury. I was really, really upset and lived in that denial, non-acceptance space for probably 2 days, and I complained to anyone who would listen. But then I realized—I have some control over this. There are things I can do. This isn’t something I have to get surgery on, and it’s not career-ending. Recognizing that I had some autonomy was huge. The more I leaned into rehab, the quicker I’d get back out there. Sitting around and complaining wasn’t going to change anything—it would probably just make me feel worse.

Meanwhile, in her recounting of adversity and resilience, **Eloise** (LQ; 19; Ice Hockey) spoke to common suffering and humanity among athletes, and how a lack of communication can make adversity and suffering feel more personal and isolating than it actually is:

It definitely wasn't just me feeling that way. I know so many other girls were in that position that year—at other schools and my own—feeling the same way, and some probably had it even worse. I didn't know because I wasn't reaching out to them, and they probably weren't reaching out to each other either.

Overall, participants highlighted the role of self-awareness and relational connection in resilience. Those who felt disconnected from their bodies, emotions, or support systems struggled to navigate stress effectively, often experiencing emotional suppression or isolation. In contrast, athletes who developed attunement, by recognizing their needs, strengths, and limitations, reported greater confidence and adaptability. This shift fostered healthier decision-making and stronger interpersonal relationships, reinforcing resilience as both an internal and external process. By integrating self-regulation with social support, participants emphasized that resilience is not solely an individual trait but one that thrives through both personal insight and connection with others.

#### Avoidance to acceptance of adversity

A second spectrum described by participants was the evolving way athletes acknowledged discomfort, challenges and setbacks. Participants associated low resilience with an avoidance of adversity and went on to explain that resilient people welcome adversity, perceiving it as an inevitable and necessary part of growth. Adversity avoidance manifested as denial of injuries, resistance to feedback, and an unwillingness to try new things to circumvent discomfort and/or failure. For example, **Alice** (UQ; 21; Soccer) provided an account for how harmful notions about resilience reinforced avoidant behavior in her injury recovery:

A lack of resilience is when someone is unable to accept where they are, so they will avoid the situation that they're faced with. A lot of times, I would just sweep injuries under the rug. I did that a lot with concussions. It wasn't necessarily that I didn't want to deal with them, but that I thought it was not resilient to take care of an injury.

Meanwhile, **Courtney** (UQ; 20; Ice Hockey) reflected on interpersonal difficulties with teammates and coaches, and how her avoidance impacted conflict resolution and team dynamics:

I could have been much more positive and more open to hearing what other people had to say—their sides of the story. I realize now that I spent so much time focusing on how no one was listening to my side. Instead of engaging, I just shut down completely. I closed myself off, didn't want to talk about it, and avoided talking to anyone. I definitely should have approached the situation with a more positive attitude, recognizing it as a valuable learning moment. People aren't going to always understand you, where you're coming from or, what you're trying to say but you have to be able to work through these things.

Although several participants spoke to the avoidance of adversity, more participants spent time speaking to the acceptance of and willingness to engage with adversity. Acceptance manifested as an openness to trying new things, a willingness to compromise and adapt their coping strategies, an appreciation for one's current circumstances, and a belief that adversity changes you for the better. These manifestations are exemplified by **Olivia's** (LQ; 30; Boxing) conceptualization of resilience:

To me, resilience means being able to approach any situation, obstacle, or adversity without immediately giving up or being deterred. It's about facing challenges—maybe even welcoming them—and finding a way to either accept what can't be changed or move through and past it, depending on the situation. I'd describe resilience as an effective way to exist as a human. Life will always have ups and downs, with winding roads rather than a straight, clear path. Resilience is about feeling confident in your ability to handle whatever comes your way—whether it's something familiar or entirely new—and trusting yourself to assess and navigate the obstacles ahead.

Overall, participants described resilience as the ability to face challenges rather than avoid them. Avoidance, through denial, suppression, or disengagement, was seen as an unsustainable strategy that hindered growth. In contrast, those who embraced adversity as part of the process reported greater adaptability, emotional endurance, and a mindset shift that framed setbacks as learning opportunities rather than threats.

#### Maladaptive to adaptive coping during adversity

The third spectrum described by participants pertained to how athletes approached and navigated adversity. On one hand, participants recounted several maladaptive coping strategies that they identified as providing short-term relief but ultimately hindered their growth, performance and/or health (e.g., disengagement from self-care and help seeking, perpetuating negativity among teammates and social withdrawal). On the other hand, participants spoke heavily about and emphasized the importance of active participation in overcoming adversity, and how this leads to more sustainable self-improvement and overall better health and wellbeing. For example, Ava, a 24-year-old volleyball player, explained:

I think I could have benefitted from engaging with my resources earlier or exploring different ones. I've had an incredible therapist since the bad one, but why did I keep going back to the bad one? I could have sought someone else or engaged with other professionals. I never spoke to anyone at my school because I felt it would be more harmful to my progress. I worried that if I was honest about how much I was struggling, they would tell me to stay back, and that wasn't what I wanted. So, I was afraid to use my school's resources.

I also don't think I would have downplayed my struggles as much to others. I should have been more honest, especially with my coaches—not because I wanted them to think I couldn't contribute, but because by downplaying it, I put myself through a lot of pain. I came back earlier than I should have and ended up with a hairline fracture because of it. That experience was definitely a test of patience. I learned a lot from it, but if I had exhibited more patience at the time, maybe I would have been in a different place.

Moving along this spectrum, participants recalled numerous adaptive coping strategies, including emotion regulation (i.e., *deep belling breathing; listening to and singing along to music; repeated, hard shots on goals*), constructive and de-escalating self-talk (i.e., *You have been through this before, and you will get through this again*), problem solving and goal setting (i.e., *identifying steps and possible obstacles needed to become a captain*), and, self-care and help seeking (i.e., *having interests and hobbies outside of sport; connecting with a psychologist, taking time away from the sport*), with several athletes noting the importance of trying different strategies to determine what works best for them and their situation. In one instance, **Sam** (LQ), a 32-year-old Triathlete likened these strategies to tools in a toolbox: *A part of resilience is knowing that you have all the tools in your toolbox and knowing what tool to pull out when the sink is leaking or when the electricity goes off or when you get a flat tire.*

Overall, participants highlighted the impact of coping strategies on resilience. While maladaptive coping, such as overtraining, self-criticism, or emotional withdrawal, offered temporary relief, it often led to burnout and decreased performance. In contrast, adaptive coping supported emotional regulation, problem-solving, and constructive responses to setbacks. The ability to shift toward adaptive strategies was seen as essential for long-term resilience, equipping athletes to navigate pressure and uncertainty more effectively.

### Programmatic preferences

The final theme pertained to athlete's preferences and suggestions for future resilience-based interventions and features. Participants emphasized the importance of focusing on gender-specific experiences with resilience, including what it is, how it applies both in and beyond sport, and why it is particularly crucial for women athletes. They highlighted the unique challenges women face, such as stereotypes that portray them as weaker, overly emotional, or unable to sustain long-term athletic careers. Resilience education should also address its connection to mental health and emphasize its role as a translatable skill.

Participants identified key strategies for building resilience, including shifting mindset, self-confidence, self-compassion, reflection, gratitude, mindfulness, focusing on controllable factors, social support, and reframing setbacks. They stressed the importance of normalizing emotions in sport, learning to ask for help, and setting realistic expectations rather than comparing oneself to others. Access to resources and role models, including adaptive and relatable examples of resilience, was also seen as valuable in reinforcing these concepts.

When discussing how resilience should be taught, participants cautioned against shaming, reinforcing stereotypes, oversimplifying resilience (e.g., “just bounce back”), or ignoring setbacks. They preferred in-person sessions over online formats, with opportunities for group discussions, breakout sessions, and workshops with trusted role models, sport psychologists, or relatable athletes. While online options were seen as useful for accessibility, participants valued interactive and team-based learning experiences most.

## Discussion

This qualitative study contributes to a broader research program examining multi-level risk and protective factors for resilience among women athletes, with the aim of informing resilience-based training. Guided by a socio-ecological model (42), findings are discussed across interconnected levels of influence, including societal and cultural, organizational and institutional, interpersonal, and individual domains. This structure highlights how resilience among women athletes is shaped not only by personal characteristics, but also by broader systems, structural conditions and relationships within sport.

### Societal and cultural influences on resilience

Consistent with previous commentary, participants expressed frustration with the patriarchal sports system and explained how this system, designed for men, by men, fails to prioritize or accommodate girls and women (43). Consequently, women athletes must work exponentially harder to compensate for and/or overcome subpar conditions, making success an exception rather than the norm. A key limitation of existing theoretical models for resilience in sport is their omission of patriarchal influences and the resulting gender-specific factors and stressors encountered by women (23–25). These models primarily emphasize individual traits and abilities in developing and maintaining resilience, neglecting the systemic and structural barriers that shape resilience across genders. Furthermore, existing resilience frameworks frequently overlook not only gender but also race, class, sexuality, and disability, failing to account for how these intersecting identities shape resilience and compound stress for marginalized individuals. By framing resilience as an individual responsibility, these models overlook the role of organizations, institutions, and society in reinforcing inequalities, implicitly expecting women and marginalized groups to adapt and “tough it out” rather than pushing for meaningful systemic change. To be more effective, resilience frameworks must acknowledge sociocultural influences, apply an intersectional lens, integrate gender-sensitive approaches and reframe resilience as a dynamic process shaped by broader social, political, and cultural systems and climates.

Another prominent sociocultural theme was the harmful rhetoric and misconceptions about resilience in sport, including defining resilience as ignoring or pushing through pain, measuring it by the number of adversities endured, and equating it with refusing to quit or walk away. In some instances, participants were aware of and challenged these beliefs and discourses, while others appeared to internalize them. Participants also explained that this rhetoric and these misconceptions occurred across different levels of society; however, they were most salient and impactful when they occurred in more proximal environments (e.g., team norms and culture) and interpersonal dynamics (e.g., coach-athlete relationships). In sport, these narratives are deeply embedded in training environments, where athletes are socialized to conform to a “sporting ethic”, which emphasizes sacrificing for the game and in some instances equates suffering with strength (44). While this mindset can foster short-term performance gains, it has been linked to long-term negative consequences, including injury neglect, psychological distress, and career burnout (45–47).

These resilience narratives also intersect with gendered societal expectations in sport. Research suggests that women athletes experience resilience demands differently than men, as they must navigate both the expectation to exhibit mental and physical toughness and the pressure to maintain socially acceptable expressions of femininity (48). This dual expectation can make it particularly challenging for women athletes to reject resilience misconceptions, as doing so may be perceived as either a lack of toughness or a violation of traditional gender norms. Furthermore, women athletes are evaluated through a lens of self-sacrifice, where their commitment is judged by their willingness to endure adversity without complaint (49). Such framing reinforces the idea that resilience is a test of suffering rather than a dynamic and context-dependent process. These sociocultural conditions shape not only the adversities women athletes encounter, but also the meanings they assign to resilience and the strategies they perceive as legitimate or acceptable.

### Organizational, institutional & interpersonal influences on resilience

Compared to other sociocultural agents (i.e., athletic trainers, parents), coaches were identified as key proponents of inaccurate, oversimplified, and harmful conceptualizations of resilience, and subsequently as individuals who encouraged and/or role-modeled maladaptive attitudes and coping strategies. Research into coaches' resilience and its subsequent impact on sport climates, as well as athletes' performance, health, and well-being, is growing but remains limited (50).

At the organizational and institutional level, coaches in women's sport are often required to operate within resource-constrained systems and are expected to succeed with fewer resources and less structural support. This high-pressure role has been associated with stress, emotional dysregulation, anxiety, depression, and burnout (51–55). These structural constraints shape not only coaches' own well-being, but also the environments they are able to create for athletes, and, in turn, influence the relational climates in which resilience is fostered or undermined.

At the interpersonal level, coaches occupy a particularly influential position in athletes' daily lives, shaping team norms, modeling coping behaviors, and reinforcing or challenging dominant narratives about resilience. If coaches operate with maladaptive beliefs or behaviors, the relational environment they cultivate may undermine athletes' resilience, regardless of the athletes' own efforts. When considering women athletes' resilience, a whole-systems approach must therefore account for both structural conditions and relational dynamics, including the attitudes and behaviors of coaches, athletic trainers, and directors, as they shape the culture and conditions in which athletes train and compete.

### Individual traits & skills

At the individual level, participants described resilience as a dynamic, context-responsive process shaped by a spectrum of interconnected traits and skills that interact and collectively support or hinder an athlete's ability to adapt, recover, and grow through adversity over time (30). There were several consistencies with previous qualitative studies on athletes' resilience (23–25), including themes emphasizing the acceptance of adversity as both inevitable and a growth opportunity, an attunement to and awareness of one's thoughts, feelings, and behaviors, and the importance of being proactive in working through and overcoming adversity. However, unique findings from this study are twofold: they pertain to the socioecological lens through which resilience is understood and the dynamic spectrum of traits that interact across different levels of society. Specifically, resilience at the individual level was described as existing along three key continuums: Disconnection to Attunement with Self and Others, Avoidance to Acceptance of Adversity, and Maladaptive to Adaptive Coping During Adversity. Where an athlete falls on each of these continuums, and whether they progress toward more adaptive forms of resilience or regress toward less effective patterns, is not solely an individual process but is deeply influenced by their environment. These continuums suggest that resilience is not a binary construct but a fluid positioning that shifts in response to contextual demands and available supports.

Taken together, these findings suggest that resilience interventions for women athletes must extend beyond individual skill-building. A socio-ecological perspective highlights the need for multi-level approaches that address organizational practices and policies, coach education, interpersonal dynamics, and individual coping strategies. Interventions focused solely on personal resilience may be insufficient without concurrent attention to systemic and relational influences within sport environments.

### Differences across resilience portfolios

Notably, no salient thematic differences emerged in women's perceptions and experiences of resilience based on whether they scored in the lower or upper quartiles of the CD-RISC. Although a greater proportion of participants fell within the lower quartiles, a pattern consistent with findings from the broader quantitative research program (28, 29), women across quartiles described similar socio-ecological influences shaping their responses to adversity.

However, subtle differences emerged in how resilience was conceptualized and narrated. In some instances, participants in the lower quartile described resilience in terms of the volume and severity of adversities endured, emphasizing hardship and survival. For example, Carly (LQ) framed resilience as persisting through repeated career-threatening injuries and cumulative suffering. In contrast, participants in the upper quartile more frequently described resilience as an evolving, skills-based process involving acceptance, patience, and adaptive coping. As illustrated by Alice (UQ), resilience was conceptualized less as endurance of hardship and more as a dynamic journey involving self-compassion and long-term growth. These distinctions suggest that resilience scores may be associated not with fundamentally different ecological experiences, but with variations in how adversity is interpreted, integrated, and narrated. Importantly, despite lower self-reported resilience scores, athletes across quartiles demonstrated considerable insight into the individual, interpersonal, and societal influences shaping their resilience. Furthermore, when explicitly asked whether they considered themselves resilient, all participants affirmed that they were.

This divergence between self-reported resilience scores and personal narratives suggests that conventional resilience measures may not fully capture how women athletes conceptualize and enact resilience. One possible explanation lies in gendered patterns of self-assessment. Research indicates that women tend to evaluate personal competencies more conservatively than men (56, 57). Further, women tend to exhibit greater self-criticism and a stronger tendency to internalize failure (58), which may contribute to lower self-assessments on resilience scales despite their capacity to effectively cope with adversity (59). This self-assessment gap may also be influenced by the way resilience is traditionally measured, as many scales emphasize traits that more commonly associated with traditional masculinity, including emotional suppression, independence, and perseverance under pressure. Women, however, may demonstrate resilience differently, through adaptability, relational coping, and emotional regulation, which are not always fully captured by conventional measures (60).

Taken together, these findings reinforce the need to conceptualize resilience as a dynamic, socio-ecological process rather than a static internal trait and underscore the importance of refining resilience measurement tools to better capture diverse and gender-responsive expressions of resilience in sport contexts.

### Clinical implications & future directions

The present findings extend theoretical and practical understanding of resilience in women's sport in two key ways. Theoretically, this study refines athlete resilience models by situating resilience within a socio-ecological framework and conceptualizing it as a dynamic, context-responsive process rather than a fixed individual trait. The identification of three interacting continuums, disconnection to attunement, avoidance to acceptance, and maladaptive to adaptive coping, offers a more nuanced depiction of how resilience fluctuates in response to relational and structural conditions.

Practically, the findings clarify multi-level targets for resilience-based intervention. At the individual level, interventions should prioritize acceptance-based coping, emotional regulation, and adaptive self-reflection, particularly given the convergence between the qualitative subtheme of Avoidance to Acceptance of Adversity and previously reported quantitative findings within the broader research program. Specifically, cross-sectional and longitudinal analyses from the parent quantitative studies (28, 29) demonstrated that experiential avoidance and nonacceptance are negatively associated with resilience, and that increases in acceptance over time predict higher resilience. While these quantitative results are reported elsewhere, their conceptual alignment with the present qualitative findings strengthens confidence in targeting acceptance processes in intervention development.

At the interpersonal level, coach education and relational climate-building are critical. Given the influential role of coaches in shaping resilience narratives and team norms, resilience programming should incorporate training that promotes psychologically safe, autonomy-supportive environments and challenges harmful conceptualizations equating resilience with endurance or emotional suppression.

At the organizational and societal levels, resilience interventions must move beyond trait-based approaches and address structural and cultural conditions that shape women athletes' experiences. Policy reform, equitable resource allocation, and deliberate efforts to challenge gendered expectations of suffering are necessary to create environments in which resilience can be sustainably developed rather than continuously demanded.

Finally, the discrepancy between self-reported resilience scores and athletes' lived experiences underscores the need for continued refinement of resilience measurement tools. Future research should develop and validate measures that more accurately reflect relational, adaptive, and context-sensitive expressions of resilience, particularly among women athletes. Additional qualitative research examining resilience across life domains may further clarify how sport-based resilience interacts with broader identity development and well-being.

### Strengths & limitations

This study offers several key strengths, including its focus on gender-specific and sociocultural influences on resilience, which are often overlooked in existing resilience models. By using a qualitative approach, this study captured the nuanced experiences of women athletes, providing rich, in-depth insights into how resilience is shaped by systemic barriers, interpersonal relationships, and internal psychological processes. Additionally, this study contributes to the growing body of literature advocating for resilience frameworks that move beyond individual traits and instead incorporate structural and institutional influences.

A major strength was our intentional recruitment strategy, which aimed to create an inclusive space for women from diverse racial, socioeconomic, and sexual identity backgrounds. However, despite our efforts to take an intersectional lens, we faced challenges in recruiting participants from historically marginalized groups, which limited our ability to fully capture the breadth of experiences across different identities. This underrepresentation reflects a broader issue within sport research, where systemic barriers contribute to the direct and indirect exclusion of certain populations. Research practices may fail to create safe and affirming spaces for IPOC athletes to participate and may overlook how research engagement can add burden to already demanding athletic, academic and personal workloads. As such, it is critical for future studies to implement more intentional, culturally responsive, and accessible recruitment strategies. Despite this limitation, the study's findings provide valuable groundwork for further exploration of resilience through an intersectional and systemic framework, emphasizing the need for ongoing efforts to amplify the voices of underrepresented women athletes.

## Conclusion

This study contributes to a broader research portfolio aimed at developing a comprehensive, mixed-methods understanding of resilience among women athletes. The findings underscore important limitations of traditional resilience models, particularly their failure to account for gendered stressors, structural inequities, and resilience misconceptions embedded within sport culture. By situating resilience within a socio-ecological framework, this study advances a more dynamic and contextually grounded conceptualization of resilience in women's sport.

While the study identifies key themes and practical directions for intervention, challenges in recruiting a fully intersectional sample highlight the ongoing need for more inclusive, accessible, and culturally responsive research practices. Future work should continue refining resilience frameworks to integrate sociocultural influences, advocate for systemic change, and ensure that resilience training initiatives are responsive to the diverse lived experiences of women athletes. Through sustained mixed-methods inquiry and socio-ecological framing, research can move toward more nuanced, equitable, and actionable resilience models that better support women in sport.

## Data Availability

The raw data supporting the conclusions of this article will be made available by the authors, without undue reservation.
