# Supplementary material for: “We're forced to be resilient”: exploration of prospective risk and protective factors of resilience among women athletes
Source: Front Sports Act Living. 2026 Mar 13;8:1718372. doi: 10.3389/fspor.2026.1718372 (PMC13038330; doi:10.3389/fspor.2026.1718372)
Supplement: Supplementary file 1 [file Table1.docx]

| **Theme: The system breeds adversity and resilience** | |
| --- | --- |
| **Subtheme: The patriarchy and gender equality** | |
| The patriarchy and gender inequality | An understanding that sport systems were historically built to support men and continue to perpetuate a masculine-dominated athletic perspective for women’s sports creating an unattainable standard. |
| **Subtheme: Representation and Role models** | |
| Media coverage | Access to and consistent viewership of women in sports. |
| Seeing positive role models and leaders overcome adversity | Observing role models—such as teammates, professional athletes, or mentors—successfully navigating challenges or setback |
| **Subtheme: Interpersonal dynamics and environments** | |
| Unsupportive coaches and athletic environments | Leaders who inhibit the biopsychosocial development of those who fall under their leadership. |
| Support networks | Athletic spaces where individuals feel emotionally and physically secure, valued, and empowered to express vulnerability without fear of judgment. These environments are fostered by parents, coaches, and leaders who acknowledge the multifaceted nature of the athletic experience. They actively encourage open and constructive dialogue, creating a culture of mutual respect, understanding, and growth. |
| **Subtheme: Resilience has a ripple effect** | |
| Resilience has a ripple effect | The idea that an individual’s ability to overcome challenges and persist in the face of adversity can inspire and positively influence others. This effect extends beyond the individual, fostering a supportive environment where resilience becomes a shared strength, motivating others to navigate their own struggles and create a culture of perseverance and growth. |

| **Theme: Misconceptions about resilience** | |
| --- | --- |
| **Code** |  |
| Incorrect an/or harmful stereotypes about resilience |  |
| Resilience is expected | The assumption that people are “supposed to be” resilient. |
| Resilience requires suffering | The belief that enduring hardship is a necessary component of building resilience, emphasizing the role of struggle in personal growth. |
| Walking away from adversity or negative situations can be considered quitting | Viewing the decision to disengage from challenges as a failure or lack of resilience, rather than as a strategic or self-preserving choice. |

| **Theme: Resilience is a spectrum of interconnected skills** | |
| --- | --- |
| **Subtheme: Attunement <> disconnection with self and others** | |
| **Code** | **Definition** |
| Emotion dysregulation in the face of challenges and adversity | Difficulty managing and controlling emotional responses, leading to decreased emotional energy, enthusiasm, or drive when confronted with high-pressure or challenging situations |
| Unhelpful thinking patterns | Cognitive habits that can undermine emotional well-being and performance, including, negative self-talk, all or nothing thinking, comparison making and perceived negative social evaluations. |
| Introspective reflection on one’s thoughts, feelings and behaviors | The deliberate practice of examining one’s internal experiences to enhance self-awareness and foster adaptive thinking |
| Internal locus of control | The belief and attitude that one has control over their own life outcomes, rather than external forces or circumstances being primarily responsible |
| Recognizes shared struggles and experiences among athletes | Recognizing that suffering and personal failure are part of the shared human experience, helping to combat feelings of isolation when things go wrong. |
| **Subtheme: Avoidance <> acceptance of adversity** | |
| Aversion to uncertainty, challenges, and trying new things | A tendency to avoid unfamiliar or difficult situations due to a fear of failure, discomfort, or perceived risks, driven by a desire to maintain control and predictability, which can hinder personal growth and the development of resilience. |
| Positivity in the face of adversity | The positive emotional response an athlete experiences during a challenge or setback. |
| Openness to uncertainty and trying new things | Athletes embrace unpredictability and experiment with new knowledge, skills and strategies |
| Willingness to compromise and adapt | The capacity to adjust behavior, expectations, or strategies in response to challenging situations, demonstrating flexibility and prioritization of outcomes over rigid adherence to plans |
| Appreciation for present circumstances | Gratitude and mindfulness toward their current situation, regardless of challenges they may face |
| Adversity changes you for the better | A growth mindset, appreciating past experiences and how they lead to personal improvement or transformation. |
| Openness to uncertainty and trying new things | Athletes embrace unpredictability and experiment with new knowledge, skills and strategies |
| **Subtheme:** **Maladaptive <> adaptive coping in the face of adversity** | |
| Spreading negativity to others |  |
| Social disconnection and withdrawal | Isolating yourself from others, often in response to stress, pressure, or feelings of inadequacy. |
| Not engaging in self-care and help seeking | A reluctance or avoidance in engaging in self-care activities, including reaching out to others for support, guidance, or encouragement during difficult times. |
| Perseverance in the face of adversity | The unwavering determination to persist through obstacles, setbacks, or challenges, sustained by mental toughness, grit, and a commitment to long-term goals |
| Goal and future oriented | Openness to setting specific, purposeful goals with a plan to work towards achieving them. |
| Make the most of your situation | The mindset of utilizing available resources, focusing on controllable factors, and turning challenges into opportunities for growth |
| Seeking out opportunities to thrive through adversity | Adaptability and positivity toward change, seeking growth and fulfillment even during challenging adjustments. |
| Active participant in overcoming adversity | Proactive, hands-on approach to facing and managing challenges, highlighting the individual’s role in actively working through difficult situations rather than passively enduring them. |
| Consistently shows up and puts in the work |  |
| Emotion regulation behaviors and strategies | Involves actively shaping or changing emotional experiences and responses to foster a more balanced emotional state |
| Self-care and help-seeking behaviors and strategies | A proactive approach toward overcoming challenges by reaching out for support, guidance, or resources. |
| Using constructive or de-escalating self-talk | The practice of calming oneself through deliberate, positive internal dialogue to manage stress or conflict |
| Not engaging in self-care and help seeking | A reluctance or avoidance in engaging in self-care activities, including reaching out to others for support, guidance, or encouragement during difficult times. |

**Program preferences (Objective Data Gathering)**

| **Question** |
| --- |
| If we were to teach women athletes about resilience, what do you think we should talk about? |
| What should we avoid when talking to women athletes about resilience? |
| Who is the best person to talk to women athletes about resilience? |
| What would be your preferred method for learning about resilience? For example, an online course or an in-person workshop with your teammates. |
